# Universality Classes and Information-Theoretic Measures of Complexity via Group Entropies

**DOI:** 10.1038/s41598-020-60188-y

**Published:** 2020-04-06

**Authors:** Piergiulio Tempesta, Henrik Jeldtoft Jensen

**Affiliations:** 1 Instituto de Ciencias Matemáticas, C/Nicolás Cabrera, No 13–15, 28049 Madrid, Spain; 2 Departamento de Física Teórica, Facultad de Ciencias Físicas, Universidad Complutense de Madrid, 28040 Madrid, Spain; 3 Centre for Complexity Science and Department of Mathematics, Imperial College London, South Kensington Campus, SW7 2AZ London, UK; 4 Institute of Innovative Research, Tokyo Institute of Technology, 4259, Nagatsuta-cho, Yokohama, 226-8502 Japan

**Keywords:** Information theory and computation, Statistical physics

## Abstract

We introduce a class of information measures based on group entropies, allowing us to describe the information-theoretical properties of complex systems. These entropic measures are nonadditive, and are mathematically deduced from a series of natural axioms. In addition, we require extensivity in order to ensure that our information measures are meaningful. The entropic measures proposed are suitably defined for describing universality classes of complex systems, each characterized by a specific state space growth rate function.

## Introduction

## A New Perspective on Complexity

The aim of this paper is to propose a general theoretical construction that allows us to associate a given class of complex systems with a suitable information measure adapted to this class, and expressed by an entropic functional mathematically deduced from a set of axioms, belonging to the family of group entropies^1–3^.

The main idea behind our approach is simple. In a broad range of applications, including physical and social sciences, economics and neurosciences, it is customary to use information measures based on the additive Shannon entropy (and its quantum version, the von Neumann entropy). Standard and useful indicators of complexity commonly adopted in the literature are indeed the mutual information or the relative entropy.

However, instead of using an information entropic measure defined a priori, and based on a (sometimes not fully justified) assumption about additivity, one may proceed differently. We propose to look for new information measures, written in terms of entropic functionals that are designed according to the specific properties of the system, or family of systems, under consideration.

To this aim, we shall prove a theorem that allows us to associate with a given universality class of systems a specific entropic measure, constructed in a completely algorithmic way. This measure is extensive and non-additive, and it depends explicitly on the state space growth rate function which characterizes the universality class considered. From a mathematical point of view, the derivation of each of these entropic measures is a direct consequence of an axiomatic approach, based on formal group theory. Using the group-theoretical entropic measures so defined, we shall construct a new family of information-theoretical measures of complexity.

The deep insights represented by the Tononi-Edelman-Spons Integrated Information concept is traditionally formulated mathematically in terms of sums of conditioned entropies of partitions of the considered system, in particular the human brain. However this mathematical representation does suffer from limitations^4^. The group theoretic entropies introduced in the present paper offer an alternative mathematical implementation of the original TES idea, without the need of introducing conditioning. We explain below how a new complexity measure based on group entropies can be useful to characterize the degree of entanglement of brain dynamics and, moreover gives a way to compute the capacity of a neuronal network of a certain size. In Sec. 5.2 we formulate this as a precise mathematical conjecture.

## A Group-Theoretical Approach to Information Theory: Group Entropies

Since the work of Boltzmann, perhaps the most relevant problem of statistical mechanics has been the study of the connections between the statistical properties of a complex system at a microscopic level, and the corresponding macroscopic thermodynamic properties.

The probabilistic point of view of Boltzmann, further developed by Gibbs, Planck and many others, was questioned from the very beginning by Einstein. As is well known, Einstein argued that probabilities must follow dynamics, and not vice versa. In other words, the frequency of occupation of the different regions of state space should not be given a priori, but determined from the equations of motion. A conciliation between these two points of view is still an unsolved problem: as surmised by Cohen^5^, a combination of statistics and dynamics is perhaps a necessary way out to describe the statistical mechanics of a complex system.

In this perspective, it is quite natural to hypothesize that the geometry of the state space associated with a given complex system plays a crucial role in the characterization of its main information-theoretical, dynamical and statistical features.

In this paper we shall try to shed new light on this aspect. We shall adopt in some sense an “intermediate” point of view: Indeed, instead of focusing on the dynamics of a specific system, we can consider *universality classes of systems*, defined in the following way.

Let us denote by $W=W(N)$ the state space growth rate (also called “phase space growth rate”) of a given system, i.e. the function describing asymptotically the number of allowed microstates as a function of the number of particles $N$. Since we are interested essentially in the large $N$ limit, we can always think of $W(N)$ as an integer number (i.e. we shall identify it with its integer part) for any choice of $W$. A universality class of systems is defined to be the set of all possible systems sharing the same growth rate function $W=W(N)$. For instance, many physical systems will be associated with an occupation law of the form $W(N)={k}^{N}$, $k > 1$. Other natural choices are, for instance, $W(N)={N}^{\alpha }$ and $W(N)=N!$. Generally speaking, we can partition all possible universality classes into three families: the *subexponential*, the *exponential* or the *super-exponential family* depending on whether the ratio $W(N)/{e}^{N} \sim c\in {\mathbb{R}}$, $0$ or $\infty$ in the large $N$ limit, respectively. A priori, in each of these three families there are infinitely many classes, although, not necessarily realized in terms of known complex systems. In particular, for the super-exponential class, we shall present the explicit example of the pairing model^6^, which is a generalization of the Ising model where the creation of “pairs” is allowed. This entails that the system possesses many more degrees of freedom with respect to the standard case.

We will show that by means of a group-theoretical approach, given any universality class of systems one can construct in a purely deductive and axiomatic way an entropic functional representing a suitable information measure for that class.

This approach is clearly inspired by the research on generalized entropies that in the last few decades captured a considerable interest. In particular, we will use the notion of *group entropies*, introduced in^1^, and settled in general terms in^2,3^ (see also^7^ for a recent review). Essentially, a group entropy is a generalized entropy that has associated a group law, which describes how to compose the entropy when we merge two independent systems into a new one.

Said more formally, the physical origin of the group theoretical structure relies on the axiomatic formulation of the notion of entropy due to Shannon and Khinchin. The first three Shannon-Khinchin axioms^8–10^ represent fundamental, non-negotiable requirements that an entropy $S[p]$ should satisfy to be physically meaningful. Essentially, they amount to the following properties:

(SK1) $S[p]$ is continuous with respect to all variables ${p}_{1},\ldots ,{p}_{W}$. (SK2) $S[p]$ takes its maximum value over the uniform distribution. (SK3) $S[p]$ is expansible: adding an event of zero probability does not affect the value of $S[p]$.

However, although necessary, these properties are still not sufficient for thermodynamical purposes. Indeed, we need another crucial ingredient: *composability*^11^. In^2,3^, this property has been reformulated and related to group theory as follows.

## The Composability Axiom

An entropy is said to be *composable* if there exists a function $\Phi (x,y)$ such that, given two statistically independent subsystems $A$ and $B$ of a complex system, $S(A\times B)=\Phi (S(A),S(B))$, when the two subsystems are defined over *any arbitrary probability distribution* of ${{\mathscr{P}}}_{W}$. In addition, we shall require that:

(C1) Symmetry: $\Phi (x,y)=\Phi (y,x)$. (C2) Associativity: $\Phi (x,\Phi (y,z))=\Phi (\Phi (x,y),z)$. (C3) Null-Composability: $\Phi (x,0)=x$.

Observe that the requirements $(C1)$–$(C3)$ are fundamental ones: they impose the independence of the composition process with respect to the order of $A$ and $B$, the possibility of composing three independent subsystems in an arbitrary way, and the requirement that, when composing a system with another one having zero entropy, the total entropy remains unchanged. In our opinion, these properties are also “non-negotiable”: indeed, no thermodynamics would be easily conceivable without these properties. From a mathematical point of view, the properties above define a *group law*. In this respect, the theory of formal groups^12–14^ offers a natural language in order to formulate the theory of generalized entropies. Notice that the above construction define a full group, since the existence of a power series $\phi (x)$ such that $\Phi (x,\phi (x))=0$ (i.e. the “inverse”) is a consequence of the previous axioms^13,15^. Let ${\{{p}_{i}\}}_{i=1,\cdots ,W}$, $W\ge 1$, with ${\sum }_{i=1}^{W}{p}_{i}=1$, be a discrete probability distribution; the set of all discrete probability distributions with $W$ entries will be denoted by ${{\mathscr{P}}}_{W}$.

### Definition 1.

A group entropy is a function $S:{P}_{W}\in {{\mathbb{R}}}^{+}\cup \{0\}$ which satisfies the axioms (SK1)-(SK3) and the composability axiom.

For recent applications of the notion of group entropy in Information Geometry and the theory of divergences^16^, see^17^.

## Results

### The extensivity requirement

Our approach is crucially based on the following extensivity assumption.

**Requirement [ER]**. *Given an isolated system in its most disordered state (the uniform distribution), the amount of its disorder increases proportionally to the number* $N$ *of its constituents*.

In other words, we shall require that, if $S$ is an information measure of order/disorder for that system, we must have $S(N)/N=const$. A weaker condition, suitable for macroscopic systems, is the asymptotic condition

1 $${{\rm{lim}}}_{N\to \infty }\frac{S(N)}{N}=const.$$

We stress that in this paper we are not considering thermodynamics, but a purely information-theoretical context. We shall assume the extensivity requirement and construct *entropic* information measuresbased on group entropies, namely a class of generalised entropies constructed axiomatically from group theory.

For entropic functionals, the requirement ER is intimately related with thermodynamic *extensivity*. Indeed, a fundamental requirement, pointed out already by Clausius, is that thermodynamic entropy, *as a function of N*, must grow linearly in $N$ in the thermodynamic limit when $N\to \infty$. It is immediately seen from the classical relation $S={k}_{{\mathscr{B}}}{\rm{ln}}\,W$, valid in the case of equal probabilities, that Boltzmann’s entropy is extensive for the universality class $W(N) \sim {k}^{N}$, which typically contains ergodic systems. However, the Shannon entropy $S=-{\sum }_{i=1}^{W}{p}_{i}\,{\rm{ln}}\,{p}_{i}$ may not be extensive over other universality classes. Therefore, in order to satisfy the ER, it appears clear that new entropic functionals should be found.

A crucial problem emerges naturally: given a complex system, how can one associate with it a meaningful information measure? This is the main question we address in this paper. We will prove that, surprisingly, there is a possible answer, simple and deductive.

Our main result is indeed the following: *For any universality class of systems there exists a related information measure satisfying the axioms (SK1)–(SK3), the composability axiom and requirement ER*.

Consequently, we shall propose a deductive construction of a group entropy associated with a given universality class. At the heart of this construction, there is a very simple idea: an admissible entropic information measure possesses an intrinsic group-theoretical structure, responsible of essentially all the properties of the considered entropy. This structure, provided by a specific group law, comes from the idea of allowing the composition of statistically independent systems in a logically consistent way in the case of independent systems (see also^18^ for a related discussion).

### A dual construction of entropies

Let $G\left(t\right)={\sum }_{k=1}^{\infty }{a}_{k}\frac{{t}^{k}}{k}$ be a real analytic function, where ${\{{a}_{k}\}}_{k\in {\mathbb{N}}}$ a real sequence, with ${a}_{1}=1$, such that the function ${S}_{U}:{{\mathscr{P}}}_{W}\to {{\mathbb{R}}}^{+}\cup \{0\}$, defined by

2 $${S}_{U}({p}_{1},\ldots ,{p}_{W}):=\mathop{\sum }\limits_{i=1}^{W}{p}_{i}\hspace{2.84526pt}G\left({\rm{ln}}\,\frac{1}{{p}_{i}}\right),$$

is a concave one. This function is the *universal-group entropy*. The two most known examples of entropies of this class are the Boltzmann-Gibbs entropy ${S}_{BG}[p]={\sum }_{i=1}^{W}{p}_{i}{\rm{ln}}\,\left(\frac{1}{{p}_{i}}\right)$ and the Tsallis entropy ${S}_{q}=\frac{1-{\sum }_{i=1}^{W}{p}_{i}^{q}}{q-1}$^19^. Essentially all the entropic functionals known in the literature are directly related with the class (2). The composability axiom widely generalizes the fourth Shannon-Khinchin axiom. Needless to say, when $\Phi (x,y)=x+y$, we get back the original version of the axiom (SK4), which states the additivity of the Boltzmann entropy with respect to the composition of two statistically independent subsystems. For $\Phi (x,y)=x+y+(1-q)xy$, we have the composition law of ${S}_{q}$ entropy, and so on.

The composability property, if required for any choice of the probability distributions allowed to $A$ and $B$, is a nontrivial one. A theorem proved in^20^ states indeed that in the class of trace-form entropies only the entropies ${S}_{BG}$ and ${S}_{q}$ are composable (uniqueness theorem).

However, the remaining cases can be at most *weakly composable*: the group law $\Phi (x,y)$ is defined at least over the uniform distribution. This case is especially important for thermodynamics, but it is not sufficient to cover many other physical situations.

The above discussion motivates the study of a family of entropies which are not in the trace-form class. In^3^, the family of $Z$-entropies has been introduced. They generalize both the Boltzmann and the Rényi entropies and are *strongly composable*.

An important result of formal group theory, states that there exists a power series of the form $G(t)=t+{a}_{2}/2{t}^{2}+\ldots$ such that, given a power series $\Phi (x,y)$ that satisfies the properties $(C1)$-$(C3)$, it can be represented in the form (See SM)

3 $$\Phi (x,y)=G({G}^{-1}(x)+{G}^{-1}(y)).$$

The general form of the non-trace-form entropies we wish to consider is given by

4 $${Z}_{G,\alpha }({p}_{1},\ldots ,{p}_{W})\,:=\frac{{{\rm{ln}}}_{G}({\sum }_{i=1}^{W}{p}_{i}^{\alpha })}{1-\alpha },$$

where ${{\rm{ln}}}_{G}(x):=G({\rm{ln}}\,(x))$ is a generalised logarithm and $\alpha$ is a real parameter. When $0 < \alpha  < 1$, the ${Z}_{G,\alpha }$ entropy is concave; when $\alpha  > 0$, is Schur-concave. Precisely, a generalised group logarithm is a continuous, concave, monotonically increasing function ${{\rm{ln}}}_{G}:{\mathbb{R}}\to {\mathbb{R}}$, possibly depending on a set of real parameters, satisfying a functional equation (a group law). It can be considered to be a deformation of the standard Neperian logarithm (See SM).

Throughout this paper, we shall put the Boltzmann constant ${k}_{{\mathscr{B}}}=1$.

### The main reconstruction theorem: From state space to group entropies

The following theorem formalizes in a rigorous way, completes and unifies several prior ideas already presented in a heuristic way in previous papers of ours^3,7^. However the main result is new: a direct formula simply expressing a group entropy as a function of the state space growth rate. Here we shall replace $N$ with a continuous variable $x$, which interpolates the discrete values of $N$. Also, we shall introduce a sufficiently regular interpolating function ${\mathscr{W}}={\mathscr{W}}(x)$, such that ${\mathscr{W}}(x){| }_{x=N}=W(N)$. In other words, ${\mathscr{W}}(x)$ is a “continuous version” of the state space growth rate. For the purposes of this article, from now on we shall also require that ${\mathscr{W}}$ is a monotonic, strictly increasing function of class ${C}^{1}({{\mathbb{R}}}^{+})$, taking positive values over ${{\mathbb{R}}}^{+}$. Hereafter, $W$ will denote the integer part of ${\mathscr{W}}$ (Usually, in the literature $W(N)$ and ${\mathscr{W}}(N)$ are identified for simplicity of notation. For large values of $N$, the discrepancy between the two values is numerically very small).

#### **Theorem 1.**

 *Let* ${\mathscr{W}}$ *be a state space growth function, corresponding to a given universality class of statistical systems. Then there exists a unique entropy in the* $Z$-*class which satisfies the extensivity requirement. This entropy is given by*

5 $${Z}_{G,\alpha }({p}_{1},\ldots ,{p}_{W})=\frac{1}{{({{\mathscr{W}}}^{-1})}^{{\prime} }(1)}\left({{\mathscr{W}}}^{-1}\left((\mathop{\sum }\limits_{i=1}^{W}{p}_{i}^{\alpha }{)}^{\frac{1}{1-\alpha }}\right)-{{\mathscr{W}}}^{-1}(1)\right),$$

with $\alpha  > 0$ and it is assumed ${({{\mathscr{W}}}^{-1})}^{{\prime} }(1)\ne 0$.

*Proof*. Let us assume that the asymptotic behaviour of ${Z}_{G,\alpha }(p)$, for large values of $N$, is given by

6 $${Z}_{G,\alpha }[{\mathscr{W}}(N)]=\lambda N+\mu $$

for suitable constants $\lambda$ and $\mu$ (which a priori could depend on thermodynamic variables, but not on $N$). This condition obviously implies extensivity, namely Eq. (1). The more general form (6), which also includes the constant $\mu$ is introduced for further convenience.

Any generalised logarithm is represented in the form (see SM)

7 $${{\rm{ln}}}_{G}(x)=G({\rm{ln}}\,x),$$

for an invertible function $G$, whose behaviour around zero is given by

8 $$G(t)=t+O({t}^{2})$$

which also implies the property

9 $$G(0)=0.$$

This function is the “group exponential” that defines the composition law of the entropy by means of relation (3). Therefore from Eqs. (4), (6), we obtain

10 $$G({\rm{ln}}\,{\mathscr{W}}{(N)}^{1-\alpha })=(1-\alpha )\lambda N+\Gamma ,$$

where $\Gamma =(1-\alpha )\mu$ is another constant. Hence the relation between the state space growth rate and the group exponential is explicitly given by

11 $${\mathscr{W}}(N)={({e}^{{G}^{-1}[(1-\alpha )\lambda N+\Gamma ]})}^{\frac{1}{1-\alpha }}.$$

Let

$$t\,:\,={\rm{ln}}\,{\mathscr{W}}{(N)}^{1-\alpha }\;\iff \;N={{\mathscr{W}}}^{-1}({e}^{\frac{t}{1-\alpha }}).$$

We obtain from Eq. (10)

$$G(t)=\lambda (1-\alpha ){{\mathscr{W}}}^{-1}({e}^{\frac{t}{1-\alpha }})+\Gamma ,$$

which implies, using relation (9)

$$G(0)=\lambda {{\mathscr{W}}}^{-1}(1)+\Gamma =0\;\iff \;\Gamma =-k(1-\alpha ){{\mathscr{W}}}^{-1}(1).$$

Consequently we get

12 $$G(t)=\lambda (1-\alpha )\left({{\mathscr{W}}}^{-1}\left({e}^{\frac{t}{1-\alpha }}\right)-{{\mathscr{W}}}^{-1}(1)\right).$$

In order to fix the constant $\lambda$, it is sufficient to observe that the condition (8) implies that

$$\lambda =\frac{1}{{({{\mathscr{W}}}^{-1})}^{{\prime} }(1)},$$

where by assumption ${({{\mathscr{W}}}^{-1})}^{{\prime} }(1)\ne 0$. In other words, the group exponential can be uniquely determined by the specific choice of a universality class of systems.

From the explicit expression of this function we can reconstruct the entropy we are looking for:

13 $${Z}_{G,\alpha }[p]=\frac{{{\rm{ln}}}_{G}({\sum }_{i=1}^{W}{p}_{i}^{\alpha })}{1-\alpha }=\frac{1}{{({{\mathscr{W}}}^{-1})}^{{\prime} }(1)}\left({{\mathscr{W}}}^{-1}\left(({\sum }_{i=1}^{W}{p}_{i}^{\alpha }{)}^{\frac{1}{1-\alpha }}\right)-{{\mathscr{W}}}^{-1}(1)\right).$$

The constant appearing in the r.h.s. of the previous formula guarantees that the entropy vanishes over a “certainty state”, namely for a distribution where $\exists \,i$ such that ${p}_{i}=1,$ ${p}_{j}=0,$ $j\ne i.$

Observe that for $\alpha  > 1,$ then ${Z}_{G,\alpha }[p]$ is still a $\mathrm{non} \mbox{-} \mathrm{negative}$ function.

The case $\alpha \to 1$ is admissible and interesting by itself, and leads us to

$${Z}_{G,1}[p]=\frac{1}{{({{\mathscr{W}}}^{-1})}^{{\prime} }(1)}({{\mathscr{W}}}^{-1}(\exp \left({S}_{BG}[p]\right)-{{\mathscr{W}}}^{-1}(1)),$$

where ${S}_{BG}[p]$ is the Boltzmann-Gibbs entropy. To conclude the proof, one can ascertain that since ${Z}_{G,\alpha }[p]$ is the composition of a strictly increasing function with a function which is strictly Schur-concave for $\alpha  > 0$, then it is still strictly Schur concave in the same interval (see e.g.,^21^ page 89 and^22^). This property is sufficient for satisfying the maximum entropy axiom. $\square$

**Remark 1**. *The entropy* ${Z}_{G,\alpha }$ *can also be expressed up to a multiplicative constant, in the form* ${Z}_{G,\alpha }({p}_{1},\ldots ,{p}_{W})=k\left({{\mathcal{W}}}^{-1}\left((\mathop{\sum }\limits_{i=1}^{W}{p}_{i}^{\alpha }{)}^{\frac{1}{1-\alpha }}\right)-{{\mathcal{W}}}^{-1}(1)\right),$ *which does not require the condition* ${({{\mathscr{W}}}^{-1})}^{{\prime} }(1)\ne 0$. *This formulation is consistent with the possibility of changing the units of thermodynamic quantities*.

**Remark 2**. *In many applications, the growth function* ${\mathscr{W}}$ *is convex in its domain*. *Then the resulting*  ${Z}_{G,\alpha }[p]$  *in particular is concave in a suitable finite interval of values of* $\alpha  > 0$, *as in the general construction of* ^3^. *When dealing with different regularity properties*, *for instance in the case of complex systems whose number of degrees of freedom is monotonically decreasing as a function of the number* $N$*of particles*, *the previous construction should be properly modified*.

#### The group-theoretical structure associated with W(N)

By construction, the following important property holds.

##### Proposition 1.

 *Let* ${\mathscr{W}}={\mathscr{W}}(x)$ *be a state space growth function. The corresponding entropy*  ${Z}_{G,\alpha }$ (5) *is strictly composable, namely*

$${Z}_{G,\alpha }(A\times B)=\Phi ({Z}_{G,\alpha }(A),{Z}_{G,\alpha }(B))$$

*for all complex systems* $A$ itand $B$, *where the group law* $\Phi (x,y)$ *associated can be written in terms of* ${\mathscr{W}}$ $as$

14 $$\phi (x,y)=\lambda \left\{{{\mathscr{W}}}^{-1}\left[\right.{\mathscr{W}}\left(\frac{x}{\lambda }+{{\mathscr{W}}}^{-1}(1)\right){\mathscr{W}}\left(\frac{y}{\lambda }+{{\mathscr{W}}}^{-1}(1)\right)\left]\right.-{{\mathscr{W}}}^{-1}(1)\right\},$$

where $$\lambda =\frac{1}{{({{\mathscr{W}}}^{-1})}^{{\prime} }(1)}$$.

*Proof*. Let us introduce the function

15 $$\chi (x):=\frac{1}{1-\alpha }G\left(\right.(1-\alpha )x\left)\right.,$$

where $G(t)$ is given in Eq. (12). We observe that

$$\begin{array}{rcl}{Z}_{G,\alpha }(A\cup B) & = & \frac{G\left({\rm{ln}}\,\left(\sum _{i,j}{({p}_{ij}^{A\cup B})}^{\alpha }\right)\right)}{1-\alpha }\\  & = & \frac{G({\rm{ln}}\,\left(\sum _{i}{({p}_{i}^{A})}^{\alpha }\right))+{\rm{ln}}\,\left(\sum _{j}{({p}_{j}^{B})}^{\alpha }\right)}{1-\alpha }\\  & = & \frac{G\left({G}^{-1}((1-\alpha ){Z}_{G,\alpha }({p}_{i}^{A}))+{G}^{-1}\left((1-\alpha ){Z}_{G,\alpha }({p}_{j}^{B})\right)\right)}{1-\alpha }\\  & = & \Phi ({Z}_{G,\alpha }(A),{Z}_{G,\alpha }(B))\end{array}$$

where $\Phi (x,y)=\chi ({\chi }^{-1}(x)+{\chi }^{-1}(y))$. Substituting the expression (15) of $\chi (x)$ we arrive at formula (14).

#### Universality classes and group entropies

An interesting question should be addressed, namely the uniqueness of the entropies defined by means of the previous construction. Certainly, we can derive other, more complicated group entropies with similar properties. Precisely, some theorems proved in^23^ and^17^ show the existence of a new, huge class of group entropies obtained by combining group entropies, of the general form

16 $$Z(p):\,=\zeta \left({S}_{1}(p),\ldots ,{S}_{l}(p)\right),$$

where ${S}_{i}(p)$ are group entropies and $\zeta :{{\mathbb{R}}}^{l}\to {\mathbb{R}}$ is a suitable function. In particular, given a group-theoretical structure, one can associate with it a family of group entropies sharing the same group structure.

However, the univariate representative (4) presents the advantage to be the “simplest” functional form within the “orbit” of a given group structure. At the same time, the $Z$-family defined in Eq. (4) is, in a sense, complete: indeed, as we have just proved under mild hypotheses, for a given universality class there exists a representative in the $Z$-family playing the role of admissible entropy. Therefore, Theorem 1 solves completely the problem of determining an entropy suitable for an arbitrary universality class. It represent a conceptually and practical powerful tool for constructing infinitely many new entropic functionals (all of them group entropies) tailored for complex systems, emerging from very different contexts: physics, social sciences, etc. Rather than an “universal entropy”, valid for any possible complex system, we have, what may be considered more reasonable, a *specific* entropy, unique in the $Z$-class, for each given universality class of systems. Different classes of entropies correspond to sub-exponential, exponential and super-exponential state space growth rates $W(N)$ were discussed in^7^.

In this respect, we point out that the super-exponential case $W(N) \sim {N}^{\gamma N}$ was already studied in some detail from the viewpoint of thermodynamics in^6^ for the so called pairing model in which each component can occupy one of two states, like in the usual Ising model; in addition to the ${2}^{N}$ states produced by Cartesian combination of the single particle states, the components may also form paired states resulting in a super exponential growth rate $W(N)$. Ref. ^6^ lists other examples of systems with a faster-than-exponential state space growth rate, namely the number of strategies in history dependent games, the space of directed adjacency matrices and the number of adjacency matrices for networks where each of $N$ nodes has exactly one edge towards any of the $N$ nodes.

The case of exponentially growing state spaces corresponds of course to the systems usually considered in statistical mechanics such as Ising models where $W(N)={2}^{N}$ or $q-$state Potts models with $W(N)={q}^{N}$.

Finally, we mention that three concrete examples of cases with sub-exponential state space growth rates are given in^24^: A super-diffusive process, a strongly restricted spin system on a network and restricted binary processes. The entropies introduced by Tsallis^11^ were designed for describing a class of models (with $W(N)={N}^{\alpha }$) belonging to the sub-exponential family.

The search for a mathematical basis of the phenomenology observed for networks as for instance in^25^ is of great interest and widely discussed in the literature, see e.g.^26,27^. The application of the approach described in this paper to network theory would start from the classification of networks in terms of the number $W(N)$ of possible microstates, which will be equivalent to the growth rate $W(N)$ of the relevant space of allowed $N\times N$ adjacency matrices. Next, to extract the network statistics of interest one could generalise to the group theoretic entropies the discussion presented in^28^ by Park and Newman, based on the Shannon entropy. For example, this can be achieved by means of the following arguments. First, one may determine the probability measure on the set of adjacency matrices ${\bf{A}}$ by use of the maximum entropy principle (under suitable constraints)^29^ applied to the entropy corresponding to the given $W(N)$. This give the probability measure $p({\bf{A}})$ on the $W(N)$ possible adjacency matrices ${\bf{A}}$, if appropriate constraints specific to the situation can be formulated^30^. Once $p({\bf{A}})$ is given, the degree distribution ${p}_{deg}(k)$ can now be extracted from the relation

17 $${p}_{deg}(k)=\sum _{{\bf{A}}}\left[p({\bf{A}}){\sum }_{i=1}^{N}\delta (\mathop{\sum }\limits_{j=1}^{N}{A}_{ij}-k)\right],\ \ k=0,1,\ldots ,N-1$$

where $\delta$ here denotes the Kronecker delta: $\delta (0)=1$ and $\delta (n)=0$ for $n\ne 0$. At this stage, it is not clear how to formulate constraints that represent the growth mechanisms such as the ones used in the Albert-Barabasi’s model^31^ or in the recent study in^32^. The authors of^32^ parametrize the simulated degree distributions in terms of q-exponentials and interpret this as a consequence of the fact that the Tsallis q-entropy maximised under the normalisation and average constraints leads to q-exponentials.

However, following standard statistical mechanics the entropy is a functional on the probability space of the microstates of a given system^29^. As is well known, in Boltzmann-Gibbs statistics the maximisation^33^ produces the Boltzmann weight ${p}_{i}$ of a microstate $i$ with energy ${E}_{i}$ as being proportional to $\exp (-\beta {E}_{i})$ where $\beta$ is the inverse temperature. For instance, one usually derives the Maxwell distribution of velocities in a gas by integration over the ${p}_{i}$ weights. The Maxwell distribution is not obtained directly by maximising the Boltzmann-Gibbs entropy. In analogy to the canonical ensemble one could let the total number of links ${T}_{{\bf{A}}}={\sum }_{i,j}{A}_{i,j}/2$ of a given network ${\bf{A}}$ play the role of energy and maximise the group entropy under the constraint that the stochastic variable ${T}_{{\bf{A}}}$ assumes a certain average value $\bar{T}$ by maximising the functional

18 $$J=S({p}_{{\bf{A}}})+{\lambda }_{1}\left(\sum _{{\bf{A}}}{p}_{{\bf{A}}}-1\right)+{\lambda }_{2}\left(\sum _{{\bf{A}}}{p}_{{\bf{A}}}{T}_{{\bf{A}}}-\bar{T}\right).$$

As discussed in^6^, in the context of Hamiltonian systems, this leads to a q-exponential distribution ${e}_{q}(x)$

19 $${p}_{{\bf{A}}}({T}_{{\bf{A}}})={e}_{q}({T}_{{\bf{A}}}).$$

From Eq. (17) we are now able to obtain the degree distribution for an ensemble of networks with a given average total number of links by substituting the expression for ${p}_{{\bf{A}}}$.

Although, by means of Theorem 1, we are able now to construct entropies *for very many universality classes*, it is usually difficult to determine the state space growth rate for a specific system. Thus, in Sec. 5.2 below we shall introduce the information measure $\Delta$ which, from experimental estimates of probability measures allows us to discriminate different functional forms of $W(N)$; from them the group entropic form relevant for the given system can be then deduced via Theorem 1. In this way one is also able to obtain information about the nature of the correlations in the system.

Consequently, our next step will be to construct an information measure associated to each of these new entropies.

All the previous discussion can be summarized as follows: Given an universality class of systems whose state space growth rate is assigned, we are able to construct an entropic functional which is extensive for $N\to \infty$, according to the classical principles of thermodynamics, and the requirements of large deviation theory^34^. Also, the entropy in Eq. (5) satisfies the first three SK axioms and is composable, with group law given by the relation (3).

Though each entropy of the class ${Z}_{G,\alpha }$ is extensive in the specific class corresponding to a given state space growth rate ${\mathscr{W}}(N)$, in general it may not be so if applied to systems with a different functional dependence of ${\mathscr{W}}$, i.e. having other occupation laws at the equilibrium. This is certainly not surprising, since it is also the behaviour of the standard Boltzmann-Gibbs entropy.

#### Explicit illustrative example

It is possibly useful to include, for completeness, a brief but detailed step by step explanation of how the above general procedure for determining the entropy works for a specific model of a complex system. To this aim, we will use the Pairing Model discussed in^6^. The model represents a generalised version of the Ising model. As is well known, the Ising model has $W(N)={2}^{N}$ states, since it consist of $N$ particles, each of which can be in one of two states. In order to model that new emergent states can be created by the interactions in complex systems, the Pairing Model assumes that any two particles may combine to form a paired state. These additional states increase the available number of states strongly; $W(N)$ is now characterised by the functional equation

20 $$W(N+1)=2W(N)+NW(N-1),\qquad N\ge 2,$$

with the initial conditions $W(1)=2$, $W(2)=5$. The first term in the r.h.s. simply refers to the fact that when adding a new particle, the number of states will be equal to all the existing states $W(N)$, each of which can exist together with one of the two states of the added particle. The second term comes about because the added particle can enter into a paired state with anyone of the existing $N$ particles; thus this paired state can be combined with any of the $W(N-1)$ available states of the remaining $N-1$ particles. If pairing is not allowed, i.e. if one considers the usual Ising model, Eq. (20) reduces to $W(N+1)=2W(N)$, which immediately gives the exponential result $W(N)={2}^{N}$. The second term leads to the super exponential result (see^6^ Eq. (4))

$$W(N)=\frac{1}{\sqrt{2}e}{\left(\frac{N}{e}\right)}^{N/2}{e}^{2\sqrt{N}}\left(1+O\left(\frac{1}{\sqrt{N}}\right)\right) \sim {\left(\frac{N}{e}\right)}^{\frac{e}{2}\times \frac{N}{e}}={{	ilde{m N}}}^{\gamma {	ilde{m N}}}.$$

So the general form of $W(N)$ for the Pairing Model belongs to the super exponential class $W(N)={N}^{\gamma N}$, which has the inverse ${{\mathscr{W}}}^{-1}(t)=\exp [L(\frac{{\rm{ln}}\,(t)}{\gamma })]$ (see also^7^). By use of Eq. (13) above we conclude that the group entropy for the Pairing Model is of the form

21 $$S[p]=\left\{\exp \left[L\left(\frac{{\rm{ln}}\,{\sum }_{i=1}^{W(N)}{p}_{i}^{\alpha }}{\gamma (1-\alpha )}\right)\right]-1\right\}.$$

Had we excluded pairing, so confined us to the Ising case, we would have $W(N)={2}^{N}$, which is the exponential class $W(N)=\exp (N)$ and hence ${{\mathscr{W}}}^{-1}(t)={\rm{ln}}(t)$. For this functional form Eq. (13) gives us, as expected, the Rényi entropy

22 $$S[p]=\frac{{\rm{ln}}\,({\sum }_{i=1}^{W(N)}{p}_{i}^{\alpha })}{1-\alpha },$$

and as mentioned above after Eq. (13), if we let $\alpha \to 1$ we arrive at the Boltzmann-Gibbs entropy

23 $$S[p]=\mathop{\sum }\limits_{i=1}^{W(N)}{p}_{i}{\rm{ln}}\,\frac{1}{{p}_{i}}.$$

### Complexity measures from group entropies

#### Shannon-type integrated measures

The traditional approach adopted to quantify degrees of interdependence between a number of components in a complex system is based on Shannon’s entropy and investigates the difference between combinations of conditional entropies. An influential example is offered by Tononi’s Integrated Information Theory^35,36^. It suggests that consciousness can be detected from measures based on Shannon’s entropy which by decompositions analyse the relationship of the information stored in the whole brain and in some specific parts. A related very recent approach seeks to analyse self-organisation of synergetic interdependencies by a focus on joint Shannon entropies^37^. And finally, let us mention the recent Entropic Brain Hypothesis^38^, which relates the increase of consciousness to the increase of the Shannon entropy, with less emphasis on how the interdependence of the conscious state is to be characterised.

The group entropy theory suggests an alternative approach, relevant when the number of degrees of freedom $W(N)$ of the entire complex system is different from the Cartesian product of the degrees of freedom of its parts. Intuitively, one can surmise that a faster-than-exponential growth of $W(N)$ may apply to the brain. Of course we don’t know the details of the relationship between the neuronal substrate and the activities of the mind, but at an anecdotal intuitive level it seems that the mind’s virtually limitless capacity of deriving and combining associations of associations in grand hierarchical structures is an example of new emergent states added to what can be reached by Cartesian combinations. Surely, our mind is able to create by composition new emerging states from old existing concepts. Take as an example the emotional mind state induced when one listens to Bach’s Chaconne from Partita No. 2 in D minor for solo violin. It seems unlikely that this state isn’t an emergent one, far beyond the Cartesian combinations.

Similarly, $W(N)$ may very likely grow much slower that the exponential dependence of Cartesian combination, for instance when one has a highly restricted system, as for argument’s sake, a financial system under very strong regulations.

In such cases the group entropies offer a way to directly quantify the extent of systemic interdependence without going through part-wise conditioning. The composability axiom relates to how the whole of a system consisting of distinct parts differs from the system one would obtain by a simple Cartesian combination of its parts. The Shannon entropy can be thought of as directly focused on the diversity in a system and then, as a second step, one can addresses interdependence e.g. by developing various related conditioned measures. In contrast the group entropies are directly sensitive both to diversity and to interdependence when the later is sufficiently strong to make $W(N)\ne {k}^{N}$.

#### A new indicator of complexity

To be precise, consider the difference

24 $$\Delta (AB)=S(A\times B)-S(AB)=\phi (S(A),S(B))-S(AB).$$

between the entropy $S(A\times B)$ of the system constructed by Cartesian combination of parts $A$ and $B$ and the entropy $S(AB)$ of the entire complex system containing the fully interacting and interdependent parts $A$ and $B$.

This measure can be thought of as a possible generalisation of the usual mutual information and could be useful as an alternative to Tononi’s Integrated Information^35,36,39–41^ being a measure that might describe the information-theoretical properties of very entangled complex systems such as for instance neural networks.

It is important to stress that the group theoretic foundation for generalised entropies offers a consistent procedure for dealing with a system consisting of two *independent* subsystems. Of course, real systems do not consist of independent parts, but we can ensure that our entropy is mathematically sound by insisting that it is able to handle the independent case. Consider a system *A* × *B*, composed of the set of states (*a*, *b*) ∈ *A* × *B* obtained as the Cartesian product of states *a* ∈ *A* and states *b* ∈ *B*. The group entropy formalism ensures that the entropy *S*_*A* × *B*_ computed directly for *A* × *B* is equal to the one obtained by first computing *S*_*A*_ and *S*_*B*_ for the two subsystems and then combining these two entropies to get the entropy for the entire system. This simply amounts to respecting that the probabilities for the states fulfil *P*_*a,b*_ = *P*_*a*_*P*_*b*_ for the independent combination of the two subsystems, which then in terms of the entropies leads to *S*(*A* × *B*) = ϕ(*S*(*A*), *S*(*B*)).

When long range forces or other kinds of long range interdependence are at play, the system $A\times B$ will be different from the system $AB$ in which all particles from $A$ and from $B$ are allowed to interact, combine and influence in whatever way the situation allows^6^.

The complexity measure defined in Eq. (24) is a way to quantify the extent of the difference between $A\times B$ and $AB$. Since the composition law $\phi (x,y)$ in Eq. (14) assumes the same functional dependence for trace and non-trace-form entropies when expressed in terms of $W(N)$ and its inverse, we conclude that the degree of complexity and its dependence on the number of degrees of freedom is fully controlled by the functional form of $W(N)$. For simplicity consider the situation where $A=B$ and each subsystem has ${N}_{0}$ particles. Furthermore, if we restrict ourselves to the uniform ensemble ${p}_{i}=1/W$, where the extensivity property of the group entropies simply make sure that $S[p]=\lambda N$, we have explicit expressions for the complexity measure $\Delta (AA)$, which we simply denote by $\Delta ({N}_{0})$. We illustrate them in three typical examples.

(I) Algebraic – $$W(N)={N}^{a}$$:
  25 $$\Delta ({N}_{0})=\lambda ({N}_{0}+{N}_{0}+\frac{{N}_{0}^{2}}{\lambda })-\lambda ({N}_{0}+{N}_{0})={N}_{0}^{2}.$$
  We might call this the “Tsallis case”: the interdependence between particles strongly restricts the available state space. The entropy of the Cartesian combination $A\times A$ overshoots the entropy of the fully “entangled” system $AA$ by ${N}_{0}^{2}$. One may think of this as indicating that the reduction of state space involves a restrictive relation between each particle and the ${N}_{0}-1$ other particles.
(II) Exponential – $$W(N)={k}^{N}$$:
  26 $$\Delta ({N}_{0})=\lambda ({N}_{0}+{N}_{0})-\lambda ({N}_{0}+{N}_{0})=0.$$
  The Boltzmann-Gibbs case where the entire system effectively is composed of a non-interdependent set of subsystems.
(III) Super-exponential $W(N)={N}^{\gamma N}$:
  27 $$\begin{array}{rcl}\Delta ({N}_{0}) & = & \lambda \{\exp [L(2(1+{N}_{0}){\rm{ln}}\,(1+{N}_{0}))]-1\}-\lambda ({N}_{0}+{N}_{0})\\  & \simeq  & \lambda \left(\exp [{\rm{ln}}\,(2{N}_{0}{\rm{ln}}\,{N}_{0})]-2{N}_{0}\simeq 2\lambda ({N}_{0}{\rm{ln}}\,{N}_{0}-{N}_{0})\simeq 2\lambda {\rm{ln}}\,{N}_{0}!.\right.\end{array}$$
  Here we assumed ${N}_{0}\gg 1$ and made use of the fact that $L(x)\simeq {\rm{ln}}\,x$ asymptotically. The effective dependence of the complexity measure on the factorial suggests a relation to the super-exponential behaviour of $W(N)$ originating in the creation of new states by forming combinations of the particles^6^.

### The complexity of human brain: a conjecture

Returning to the question of the complexity of neural networks, we wish to point out that in principle, the indicator $\Delta ({N}_{0})$ can be used to ascertain the functional form of $W({N}_{0})$ also in the case of the human brain, for example by use of histograms constructed from time series of fMRI or EEG recordings. Of course we are not able to “separate” the brain into independent sub systems $A$ and $B$. Instead we can construct distributions for sub-parts of the brain as the marginal distributions of the entire brain. This will only be an approximation compared to entirely independent sub-parts since the marginal distributions ${p}_{A}(a)={\sum }_{b}{p}_{AB}(a,b)$ will still contain contributions from collective states spanning across $A$ and $B$ (which would not be present in $A$ if it could be isolated from $B$.) Neglecting this caveat we may proceed as follows. The histogram of the simultaneously recorded signals will give us an estimate of the joint probability density $P({x}_{1},...,{x}_{N})$ where ${x}_{1}$ up to ${x}_{N}$ are the $N$ recorded times series covering a certain connected part of the brain. We break the set of time series into two groups, each consisting of ${N}_{0}\le N/2$ time series from a spatially connected part of the brain. Let $P({x}_{1},...,{x}_{2{N}_{0}})$ characterise the “full” system $AB$ discussed above. Since we are focusing on the interdependence of brain regions one should let the $N$ probes cover a connected part of the brain. Say as an illustration, F7, F3, Fz, T3, C3 and Cz in the standard EEG map notation. The subset $A$ could consist of F7, F3 and Fz and $B$ of T3, C3 and Cz. We can then extract distributions for subsystems as the marginalised probabilities. So let our sub-systems $A$ and $B$ be given by

$$\begin{array}{rcl}{P}_{A}({x}_{1},\ldots ,{x}_{{N}_{0}}) & = & \int d{x}_{{N}_{0}+1}\cdots \int d{x}_{N}P({x}_{1},\ldots ,{x}_{N}),\\ {P}_{B}({x}_{{N}_{0}+1},\ldots ,{x}_{2{N}_{0}}) & = & \int d{x}_{1}\cdots \int d{x}_{{N}_{0}}d{x}_{2{N}_{0}+1}\cdots d{x}_{N}P({x}_{1},\ldots ,{x}_{N}),\end{array}$$

and form the Cartesian non-interacting system $A\times B$ described by

$${P}_{A\times B}({x}_{1},\ldots ,{x}_{2{N}_{0}})={P}_{A}({x}_{1}\ldots ,{x}_{{N}_{0}})\times {P}_{B}({x}_{{N}_{0}+1},\ldots ,{x}_{2{N}_{0}}).$$

We can now compute $\Delta$ in Eq. (24) and by varying the number ${N}_{0}$ of data streams included in the two sub-systems, we can check the ${N}_{0}$ dependence of $\Delta$. We conjecture that for the brain, $\Delta$ will depend like ${\rm{ln}}\,{N}_{0}!$. This corresponds to case (III) above i.e. corresponding to $W(N)={N}^{\gamma N}$. Consequently, we can state the following

**Conjecture**: The number of brain states typically grows *faster than exponentially* in the number of brain regions involved.

## Discussion

The axiomatic approach proposed in this work allows us to associate in a simple way an entropic function with an universality class of systems. In particular, the extensivity axiom selects among the infinitely many group entropies of the $Z$-class a unique functional, which possesses many relevant properties, all necessary for an information-theoretical interpretation of the functional as an information measure. The standard additivity must be replaced by a more general composability principle which ensures that, in the case of a system made by statistically independent components, the properties of the compound system are consistent with the those of its components.

Many research perspectives are worth being explored in the future. Generally speaking, our formalism allows for a systematic generalisation of a statistical mechanics description to non-exponentially growing state spaces.

For instance, we believe that group-theoretical information measures in the study of self-organized criticality could replace Shannon’s entropy in several contexts where the number of degrees of freedom grows in a non-exponential way.

We also mention that classifying complex systems without worrying about composability was done in^42^.

The analysis of time series of data, from this point of view, offers another interesting possibility of testing the present theory.

A quantum version of the present approach, in reference with the study of quantum entanglement for many-body systems represents an important future objective our our research.

Work is in progress along these lines.

## Supplementary information

Supplementary Information.
