# Supplementary material for: Universality Classes and Information-Theoretic Measures of Complexity via Group Entropies
Source: Sci Rep. 2020 Apr 6;10:5952. doi: 10.1038/s41598-020-60188-y (PMC7136250; doi:10.1038/s41598-020-60188-y)
Supplement: Supplementary file 1 — Supplementary Information. [file 41598_2020_60188_MOESM1_ESM.pdf]

Universality Classes and Information-Theoretic Measures of Complexity via Group Entropies

Piergiulio Tempesta[1] **and Henrik Jeldtoft Jensen**[2,*]

[1]Instituto de Ciencias Matemáticas, C/ Nicolás Cabrera, No 13–15, 28049 Madrid, Spain, and Departamento de Física Teórica, Facultad de Ciencias Físicas, Universidad Complutense de Madrid, 28040 – Madrid, Spain
[2]Centre for Complexity Science and Department of Mathematics, Imperial College London, South Kensington Campus, SW7 2AZ, UK and Institute of Innovative Research, Tokyo Institute of Technology, 4259, Nagatsuta-cho, Yokohama 226-8502, Japan
[*]h.jensen@imperial.ac.uk

SUPPLEMENTARY MATERIAL.

Groups and entropies: a brief summary.

For sake of completeness, in order to have a self-contained exposition, we shall review here some results concerning formal group theory and its relation with the theory of generalised entropies, following closely refs.[2] and[3].

Formal group laws

We will start by recalling some basic facts and definitions of the theory of formal groups (see[5] for a thorough exposition, and[7],[6] for a shorter introduction).

Let R be a commutative associative ring with identity, and $R\{x_1,\ x_2,..\}$ be the ring of formal power series in the variables x_1, x_2, ... with coefficients in R.

Definition 1. *A commutative one–dimensional formal group law over R is a formal power series $\Psi(x,y) \in R\{x,y\}$ such that*[4]

$$1) \qquad \Psi(x,0) = \Psi(0,x) = x,$$

$$2) \qquad \Psi(\Psi(x,y),z) = \Psi(x,\Psi(y,z)).$$

When $\Psi(x,y) = \Psi(y,x)$, the formal group law is said to be commutative.

The existence of an inverse formal series $\varphi(x) \in R\{x\}$ such that $\Psi(x,\varphi(x)) = 0$ is a consequence of Definition 1. Let $B = \mathbb{Z}[b_1, b_2, ...]$ be the ring of integral polynomials in infinitely many variables. We shall consider the series $F(s) = \sum_{i=0}^{\infty} b_i \frac{s^{i+1}}{i+1}$, with $b_0 = 1$. Let $G(t)$ be its compositional inverse:

$$G(t) = \sum_{k=0}^{\infty} a_k \frac{t^{k+1}}{k+1}, \qquad (1)$$

i.e. $F(G(t)) = t$. From this property, we deduce $a_0 = 1, a_1 = -b_1, a_2 = \frac{3}{2}b_1^2 - b_2, \ldots$. The Lazard formal group law[5] is defined by the formal power series

$$\Psi_{\mathscr{L}}(s_1,s_2) = G\left(G^{-1}(s_1) + G^{-1}(s_2)\right).$$

The coefficients of the power series $G\left(G^{-1}(s_1) + G^{-1}(s_2)\right)$ lie in the ring $B \otimes \mathbb{Q}$ and generate over $\mathbb{Z}$ a subring $A \subset B \otimes \mathbb{Q}$, called the Lazard ring L.

For any commutative one-dimensional formal group law over any ring R, there exists a unique homomorphism $L \to R$ under which the Lazard group law is mapped into the given group law (the *universal property* of the Lazard group).

Let R be a ring with no torsion. Then, for any commutative one-dimensional formal group law $\Psi(x,y)$ over R, there exists a series $\psi(x) \in R[[x]] \otimes \mathbb{Q}$ such that

$$\psi(x) = x + O(x^2), \quad \text{and} \quad \Psi(x,y) = \psi^{-1}(\psi(x) + \psi(y)) \in R[[x,y]] \otimes \mathbb{Q}.$$

The universal formal group plays the role of the general composition law admissible for the construction of the entropies of the Z-family.

We also mention that in[8] the notion of *formal rings* has been recently introduced as a natural extension of the notion of formal groups.

Generalised logarithms and exponentials from group laws

There is a simple construction allowing to define a generalised logarithm from a given group law.

Definition 2. *Let G be a series of the form (1). A generalised group logarithm is a continuous, strictly concave, monotonically increasing function $\ln_G : (0,\infty) \to \mathbb{R}$, possibly depending on a set of real parameters, such that $\ln_G(\cdot)$ solves the functional equation for the group law corresponding to G, i.e*

$$\ln_G(xy) = \Psi(\ln_G(x), \ln_G(y)) \qquad (2)$$

where

$$\Psi(x,y) = G(G^{-1}(x) + G^{-1}(y)). \qquad (3)$$

Remark 1. *For the purposes of this paper, an alternative, simple way to define a group logarithm is the following: one can introduce*

$$\ln_G(x) := G(\ln x) \ , \tag{4}$$

where $G \in C^1(\mathbb{R}_{\geq 0})$ *is a strictly increasing function, with* $G(t) = t + O(t^2)$*, taking positive values over* $\mathbb{R}^+$*. Then* $G(\ln x)$ *is at least a Schur-concave function and it satisfies eq. (2), where* $\Psi(x,y)$ *is the group law (3).*

The theory, in an abstract sense, can also be formulated in a field of characteristic zero in the class of formal power series. As is well known[5], for a 1-dimensional formal group law $\Psi(x,y)$ over a torsion-free ring, there exists a formal series $G(t)$ of the form (1) that realizes eq. (3).
By way of an example, when $\Psi(x,y) = x+y$, we have directly that $G(t) = t$ and $\ln_G(x) = \ln x$. If $\Psi(x,y) = x+y+(1-q)xy$, an associated function $G(t)$ is provided by $G(t) = \frac{e^{(1-q)t}-1}{1-q}$ and the group logarithm converts into the Tsallis logarithm

$$\ln_q(x) := \frac{x^{1-q}-1}{1-q}. \tag{5}$$

Remark 2. *If we express* $G(t)$ *in terms of the series (1), the requirement of concavity of* $\ln_G(x)$ *is guaranteed by the condition*

$$a_k > (k+1)a_{k+1} \qquad \forall k \in \mathbb{N} \qquad \text{with } \{a_k\}_{k\in\mathbb{N}} \geq 0, \tag{6}$$

which is also sufficient to ensure that the series $G(t)$ *is absolutely and uniformly convergent with a radius* $r = \infty$*.*

In other words, given a group law, under mild hypotheses we may determine a group logarithm also by means of relation (4) and the condition (6) (see[1] for a construction of group logarithms from difference operators via the associated group exponential G).

Definition 3. *The inverse of a generalised group logarithm will be called the associated generalised group exponential; it is defined by*

$$exp_G(x) = e^{G^{-1}(x)}. \tag{7}$$

When $G(t) = t$, we have back the standard exponential; when $G(t) = \frac{e^{(1-q)t}-1}{1-q}$, we recover the q-exponential $e_q(x) = [1+(1-q)t]^{\frac{1}{1-q}}$, and so on.

References

1. Tempesta, P. Group entropies, correlation laws and zeta functions. *Phys. Rev. E* **84**, 021121 (2011).
2. Tempesta, P. Beyond the Shannon-Khinchin Formulation: The Composability Axiom and the Universal Group Entropy. *Ann. Phys.* **365**, 180–197 (2016).
3. Tempesta, P. Formal Groups and Z–Entropies. *Proc. Royal Soc. A*, **472**, 20160143 (2016).
4. Bochner, S. Formal Lie groups. *Ann. Math.* **47**, 192–201 (1946).
5. Hazewinkel, M. *Formal Groups and Applications* (ed. Academic Press) (New York 1978).
6. Serre, J.–P. *Lie algebras and Lie groups*, Lecture Notes in Mathematics, 1500 (ed. Springer–Verlag) (Berlin, 1992).
7. Bukhshtaber, V. M., Mishchenko, A. S. & Novikov, S. P. Formal groups and their role in the apparatus of algebraic topology. *Uspehi Mat.Nauk* **26**, 2, 161–154, transl. *Russ. Math. Surv.* **26**, 63–90 (1971).
8. Carrasco, J. A. & and Tempesta, P., Formal rings, arxiv:1902.03665 (2019).
